# Nutritional support in sepsis: when less may be more

**DOI:** 10.1186/s13054-020-2771-4

**Published:** 2020-02-14

**Authors:** Gustav van Niekerk, Charné Meaker, Anna-Mart Engelbrecht

**Affiliations:** Department of Physiological Sciences, Stellenbosch University, Stellenbosch, South Africa

**Keywords:** Nutritional support, Permissive underfeeding, Autophagy, Sepsis

## Abstract

Despite sound basis to suspect that aggressive and early administration of nutritional support may hold therapeutic benefits during sepsis, recommendations for nutritional support have been somewhat underwhelming. Current guidelines (ESPEN and ASPEN) recognise a lack of clear evidence demonstrating the beneficial effect of nutritional support during sepsis, raising the question: why, given the perceived low efficacy of nutritionals support, are there no high-quality clinical trials on the efficacy of permissive underfeeding in sepsis? Here, we review clinically relevant beneficial effects of permissive underfeeding, motivating the urgent need to investigate the clinical benefits of delaying nutritional support during sepsis.

## Introduction

Despite sound basis to suspect that aggressive and early administration of nutritional support may hold therapeutic benefits during sepsis, recommendations for nutritional support have been somewhat underwhelming. The Surviving Sepsis Campaign recommends against early parenteral nutrition, based on studies of low to moderate quality, yet the early initiation of progressive enteral nutrition was encouraged [1]. The latest ESPEN guidelines did not attempt a meta-analysis on the efficacy of enteral nutrition (EN) versus permissive underfeeding “due to paucity of related studies”, yet advises, based on expert consensus, the initiation of “early and progressive” enteral nutritional support in sepsis without shock [2]. Similarly, based on expert consensus, ASPEN guidelines also propose the initiation of EN within 24–48 h after the diagnosis of sepsis in hemodynamically stable patients [3]. In summary, it is generally advised, based on expert consensus, extrapolation from other critical care settings, or through reference to pre-clinical findings in studies of varying quality, that early enteral nutritional support may be beneficial.

This observation raises a question: why, given the “paucity of studies”, are there no high-quality clinical trials on the efficacy of permissive underfeeding in sepsis? Indeed, initiating early parenteral nutrition has even been found to solicit detrimental effects in at least some large clinical trials [4, 5], and it has also recently been pointed out that full early nutritionals support may exert detrimental effects in a clinical setting by inhibiting autophagy [6]. One reason may be that, whereas the potential benefits of nutritional support may be obvious, it is less clear as to whether permissive underfeeding would deliver any clinical benefits. Here, we review the rationale for permissive underfeeding in the critical care setting, the physiological mechanisms implicated, and the potential therapeutic benefits which may result. Specifically, we argue that the delaying of nutritional support facilitates an elevated catabolic tone, which in turn solicits a range of clinically relevant benefits. Taken together, we argue that there are legitimate reasons to urgently investigate the potential clinical benefits of permissive underfeeding in otherwise well-nourished patients during sepsis.

## Activation of the immune system antagonises GI function

Gastrointestinal (GI) complications are common in critical care patients [7]. However, such “dysfunction” of the GI-tract can also be viewed as an extension of sickness associated anorexia (SAA)—an aspect of evolutionarily conserved sickness-related behaviour. Supporting this view, it is critical to note that the decrease in GI function is not a passive occurrence, but instead represents a detailed response that manifests under the instruction of inflammatory mediators. Indeed, inflammatory mediators have a well-established role in suppressing gastric motility. Early studies have shown that subcutaneously injected LPS resulted in the suppression of both spontaneous and bethanechol-stimulated contractions in circular smooth muscle [8]. Similarly, LPS-induced secretion of TNF in the medullary dorsal-vagal complex also contributed to gastric stasis [9]. In fact, studies have shown that various pro-inflammatory cytokines such as TNF [10], Il-1β, [11] and IFN-γ [12] directly attenuate smooth muscle contraction, thereby compromising gastric motility.

There is also evidence that inflammatory mediators may alter pancreatic exocrine function. In patients with sepsis, exocrine dysfunction seems to mirror disease severity [13], implicating inflammation in the suppression of exocrine function. It is, however, not clear how inflammatory mediators promote these changes, i.e. whether they mediate these changes directly or indirectly via their effect on the nerves innervating the pancreas. It has recently been reported that inflammatory mediators can induce ductal-to-endocrine cell reprogramming in mice, even in the absence of hyperglycaemia [14], suggesting that inflammatory mediators may supress digestion by inducing a phenotypic “switch” in exocrine cells.

Earlier studies have shown that both Il-1β and TNF inhibited gastric acid secretion by rabbit parietal cells [15]. More recently, it has also become apparent that chronic Il-1β exposure not only inhibits acid secretion, but promotes gastric atrophy by suppressing the Hedgehog signalling pathway [16]. The synthesis of bile acids (BA) also seems to be disabled by inflammatory mediators. Earlier studies have implicated the decreased expression of BA transporters at the bile canaliculi as a contributing factor to sepsis-associated cholestasis [17]. In fact, CYP7A1, the first gene in BA synthesis, is surpassed by both TNF and Il-1β [18]. Thus, both BA release and synthesis is surpassed by inflammatory mediators. In summary, inflammatory mediators not only suppress appetite, but also interrupt digestion on various levels.

Inflammatory mediators thus do not only supress appetite (i.e. SAA), but play a much more involved role in antagonising digestion. Such a comprehensive inhibition of gastric function may well represent a strategy to avoid investing resources into a system that is unlikely to be utilised during an infection. However, this observation also raises two further questions. Firstly, if inflammation inhibits GI function on numerous levels, what is the effect of enteral nutritional support? Feeding during a severe inflammatory response may represent an underappreciated cause of complications through the forced engagement of physiological processes that are not intended to be operational during an infection. Secondly, we speculate that the suppression of digestive machinery is more than just conservation of energy. Specifically, we hypothesise that the decommissioning of the GI tract is an extension of SAA and forms part of an immunological strategy to augment systemic catabolism in non-immune tissue.

## Catabolism repurposed for survival

Inflammatory mediators are potent inducers of catabolism. Indeed, cytokines have a well-appreciated role in inducing the breakdown of proteins in muscle, promoting bone resorption and also driving lipolysis in adipocytes [19]. In turn, the catabolic state also drives what has until recently been described as a manifestation of “metabolic derangements” such as the hyperglycaemia invariably observed in critical care patients. However, there is evidence to suggest that catabolism is more than just a means to an end (i.e. the liberation of metabolic substrate) but is in itself a survival strategy.

Macro-autophagy (hereafter simply autophagy) is an evolutionarily conserved catabolic process that plays an essential role in promoting cell survival [20]. Conceptually, the autophagic process consists of two major steps. Firstly, the targeted substrate must be isolated prior to catabolism; this is followed by fusion of the isolated substrate (autophagosome or amphisome—pending on the origin of the cargo) with lysosomal vesicles which subsequently degrade the vesicle’s cargo. Various different substrates are known to be targeted for lysosomal degradation. Lipophagy describes the targeting of intracellular lipid droplets for catabolism, and correspondingly, glycophagy mobilises glycogen stores [21]. These observations then implicate autophagy as a key role-player in liquidating cellular structures, thereby freeing resources for utilisation in other processes. However, autophagic machinery is also implicated in other activities.

Misfolded proteins represent a major cellular danger, as these disorganised proteins are prone to form toxic protein aggregates. Proteins may be misfolded, either because of a denaturing environment or because of direct protein damage (e.g. free radical damage). Interestingly, studies in mice show that febrile range increases in body temperature resulted in an increased expression of heat shock proteins [22]: this observation suggests that even a slight increase in temperature may result in increased protein misfolding which necessitates the increased expression of chaperones. Of note, we speculate that protein misfolding in the febrile range most likely does not present a major challenge for proteins in their native state. In other words, febrile range temperatures do not necessarily induce the denaturation of proteins already properly folded. Rather, because “the folding environment is finely tuned to the specific needs of a given cell and tissue” [23], newly synthesised proteins may fail to reach the native conformation during a febrile response. Regardless, both endoplasmic reticulum (ER) stress, as well as the cellular response to ER stress (i.e. the unfolded protein response), is believed to be activated in the critical care context, including during sepsis [24]. In this regard, a catabolic state may be protective by rendering the cell more effective in removing damaged proteins. While the proteasome may degrade misfolded proteins, aggrephagy (a specialised form of autophagy implemented in the clearance of toxic protein aggregates) would play a key role in the removal of toxic protein aggregates too large for the ubiquitin proteasome pathway. In fact, it has recently come to light that autophagy also selectively target the ER (ER-phagy) and is believed to play a key role in maintaining proteostasis during ER stress [25]. Autophagy also has a well-established role in reshaping the proteome. As an example, recent findings in cancer cells revealed an exquisite selectivity in the pool of proteins targeted for degraded and that such remodelling of the proteome may promote cell survival by attenuating inflammatory processes [26].

Though overt cell death is actually rare in sepsis, there is evidence of increased mitochondrial dysfunction [27], suggesting that mitophagy (a specialised form of autophagic digestion, which degrades mitochondria) may be critical in ensuring mitochondrial quality control during a severe infection. Indeed, mitochondrial quality control is known to play a critical role under normal physiological conditions. As an example, thyroid hormone (T3) induces an increase in oxidative phosphorylation, but also in mitophagy [28]. The reason for this seemingly paradoxical state, during which increased utilisation of mitochondrial respiration is coupled with increase mitochondrial clearance, is to improve quality control, which is necessary to maintain elevated levels of mitochondrial function [28]. Notably, mitochondrial function (e.g. oxidative phosphorylation), as well as ROS production, is enhanced by febrile range temperatures (< 40 °C) [29], suggesting that mitochondrial quality control might similarly be crucial during a febrile response. Indeed, failure to remove damaged mitochondria is also believed to increase ROS production, suggesting that enhanced mitophagy may be protective by preventing the production of ROS by defective mitochondria. In summary, mobilisation of catabolic machinery may allow a more responsive regulation of mitochondria quality, thereby avoiding excessive ROS production.

We have previously pointed out that autophagy may play a critical role in the removal of inflammogens, such as LPS, by the lysosomal enzyme, acyloxyacyl hydrolase [30]. Moreover, autophagic machinery also plays a pivotal role in pathogen clearance (known as xenophagy). Indeed, the critical role of autophagy in pathogen control is evident from the fact that viruses and bacteria have evolved numerous strategies to curtail and subvert autophagic processes [31]. As an example, it was recently shown that selective xenophagy of *Mycobacterium tuberculosis* surface protein such as Rv1468c can be targeted for ubiquitination, followed by p62 recruitment, and the subsequent delivery of the Rv1468c-ubiquitin-p62 complex to LC3-decorated autophagosomes for selective degradation [32]. Importantly, autophagy not only operates in immune cells, but also forms a key cellular response to pathogen infection in non-immune cells [33]. For example, IFN-γ treatment of hepatocytes mobilised various autophagy-related proteins that play a key role in LC3-associated phagocytosis (LAP)-like degradation of the malaria parasite [34]. However, as mentioned, pathogens have also evoked strategies to undermine and even co-opt autophagic processes for their own survival. In this regard, we have previously argued that a pre-existing catabolic state (i.e. upregulating autophagic processes) may be adaptive [35]: since degradation processes are already in full swing in cells exhibiting a catabolic state, intracellular pathogens would be confronted with a narrow window of opportunity to engage countermeasures (i.e. subverting autophagy or escaping from cellular compartments targeted for autophagic destruction). Catabolism is therefore an adaptive strategy, aimed at generating a hostile intracellular environment, thus preventing the propagation of infectious agents.

Finally, autophagy contributes to the processing and presentation of peptides on both MHC I and II. As an example, autophagy can also act as a proteasome-independent alternative pathway for the processing and loading of viral-derived peptides onto MHC I [36]. Specifically, autophagy-mediated processing may be a critical back-up during viral subversion of proteasomes [36]. Furthermore, an underexplored consequence of autophagy in epitope generation is that proteasomes and autophagy can produce different peptides from similar antigenic proteins. Peptides loaded on MHC I are typically in 8–12 amino acids in length; MHC II are between 14 and 20 [37]. This implies that the autophagic processing of proteins for presentation on MHC II may alter the immunogenicity of potentially infected cells. In support of this possibility, it was recently shown that treatment of cancer cells with IFN-γ altered the processing and length of peptides loaded on MHC proteins [38]: since IFN-γ is a potent inducer of autophagy [39], it is possible that autophagy may alter the immunogenicity of epitopes expressed by cells, rendering infected cells more visible to the immune system. In fact, studies on oncolytic adenoviruses have shown that induction and inhibition of autophagy can qualitatively impact epitope expression by altering the repertoire of peptides generated for MHC presentation [40]. Collectively, there is a clear need to investigate the immunological significance of autophagy-generated peptides for MHC complexes and the potentially altered immunogenic properties of these peptides.

Since fasting is a potent inducer of autophagy, we have previously argued that SAA ensures adequate levels of autophagic activity during an infection [35]. In this regard, nutritional support may well inhibit autophagy by elevating amino acids levels, thereby attenuating autophagy via mTOR signalling. Feeding also solicits physiological processes that supress catabolism while promoting anabolism. A classic example includes the release of insulin following a meal: insulin is a canonical inhibitor of autophagy, while catabolic hormones such as glucagon (which is suppressed by feeding) induce autophagy. Similarly, we have highlighted that a key aspect of nutritional support which is seldom addressed is the physiological response to feed-fast cycles, specifically, the signalling effects of post-prandial reabsorbed bile acids [30]. For example, secondary BAs can modulate immune function via its activity on G protein-coupled bile acid receptor 1 and the Farnesoid-X-Receptor [41]. Notably, FXR activation by BAs is also known to potently supress the transcription of key autophagic genes [42]. In addition, activation of these BA-receptors also exerts an anti-inflammatory effect by promoting a more tolerogenic phenotype in various immune cells [41].

The preceding discussion also highlights that other aspect of nutritional support besides nutrient content and suppression of catabolic processes may be impacted by nutritional support. BA release following nutritional support may also impact on immune cell function, inhibiting autophagy and vascular tone (e.g. increase splanchnic blood flow) [30]. Similarly, a recent expert consensus have pointed out the emerging role of intestinal biota in a critical care setting [43]. This raises an intriguing question: could nutritional support during sepsis exert a clinically relevant effect on the host microbiome? In a fasted state, bacteria populations can be manipulated by the host secretion of O-linked glycans to “intentionally influence this ecosystem for better health and nutrition” [44]. In a fasted state, the survival of more “domesticated” intestinal biota may be promoted by selecting the population of bacteria that are better able to survive on host-derived glycans. Furthermore, during sepsis, higher ethanol levels were observed in the urine of patients with poor prognosis [45]. Since ethanol is only derived from fermentation by gut biota (patients nutrition was controlled for 24 h), it suggests that the microbiome may impact on sepsis. It is thus clear from these observations that nutritional support may affect host-microbiome during sepsis.

**Fig. 1:**
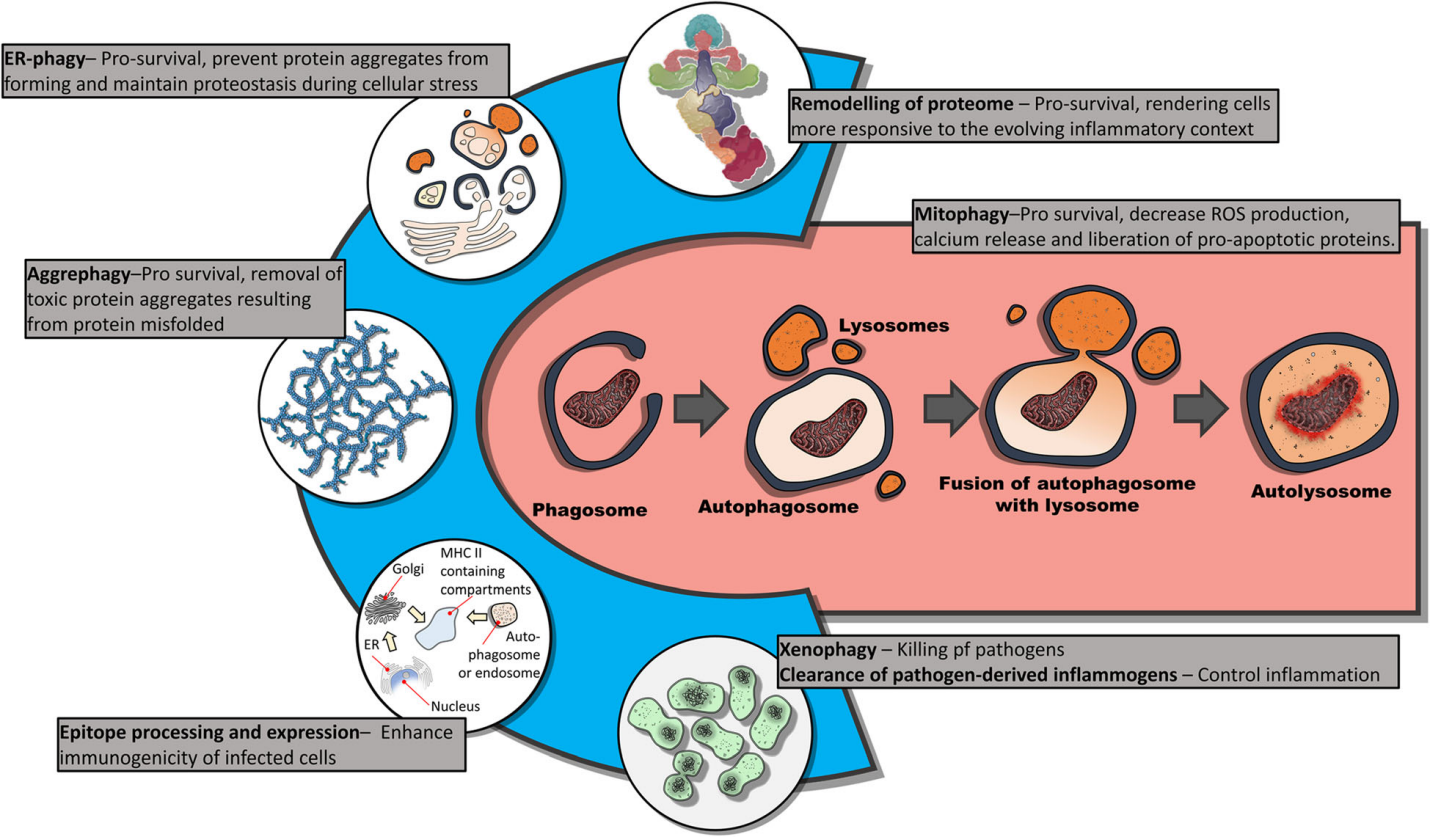
Autophagy is an evolutionarily conserved stress response that is upregulated by a range of cellular stressors, including fasting as well as various pro-inflammatory signals. In turn, this catabolic process may be dynamically repurposed to resolve a range of cellular stresses that may emerge during sepsis. This includes the removal of large protein structures as well as remodelling of the proteome to better accommodate emerging stressors faced during sepsis. ER-phagy as well as aggrephagy plays a role in preventing the accumulation of toxic protein aggregates, whereas xenophagy represents an indispensable mechanism in cell-autonomous defence against intercellular pathogens. Autophagy is also involved in the processing and presentation of both endogenous and exogenously derived epitopes, thereby playing a potential role in regulating the immunogenicity of infected cells. Autophagy also has a well-established role in reshaping the proteome. As an example, recent findings in cancer cells revealed an exquisite selectivity in the pool of proteins targeted for degradation and that such remodelling of the proteome may promote cell survival by attenuating inflammatory processes

The loss of appetite as part of sickness behaviour, coupled with the comprehensive shutdown of the gastric system, is likely an adaptive response aimed at sustaining elevated levels of catabolism and, specifically, autophagy. Remarkably, it is also worth noting that several pro-inflammatory cytokines (e.g. TNF,Il-1β, Il-6, Il-17, and IFN-γ) have been shown to regulate autophagy (reviewed [39]). Similarly, damage-associated molecular patterns such as HMGB1 [46] and various pathogen-associated molecular patterns that are recognised by respective TLRs [47] all have well-established roles in activating autophagy. The observation that SAA manifests in context of inflammatory mediators that also induce autophagy is likely no co-incidence: we argue that the innate suppression of feeding represents a tactic to synergistically converge cytokine-induced catabolism with fasting-induced catabolism, resulting in a synergistic potentiation of catabolism thereby promoting cell survival and enhanced immune function (Fig. 1).

## Conclusion

For clinicians, the term “catabolism” usually carries strong negative connotations. Remarkably, however, it is worth noting that across the animal kingdom, organisms in a non-anabolic state (e.g. spores or a dauer and pupa phase) display greater resilience to various stressors. Similarly, mice in a fasted state are far more tolerant of the toxic effects of both radiation and chemotherapy: this observation forms the basis for a number of clinical trials which evaluate the efficacy of fasting prior to receiving chemotherapy. Here, we have outlined a number of key physiological processes invoked by catabolism and provide a rational justification for the evaluation of the efficacy of permissive underfeeding during sepsis in otherwise well-nourished individuals.

## Data Availability

Not applicable.

## Abbreviations

BA: Bile acids
ER: Endoplasmic reticulum
GI: Gastrointestinal
SAA: Sickness associated anorexia
